# Clinical Impact of Macronutrients and Micronutrients: A Review of Nutritional Balance, Deficiency Disorders, and Therapeutic Applications

**DOI:** 10.7759/cureus.105305

**Published:** 2026-03-16

**Authors:** Mamellapalli Radhika, Suman Mohan, Harsimran Jit Singh, Pallavi Kadwe, Jatin Prajapati, Sohilbhai Hasanbhai Mansuri, Jagruti Jayant Sonis

**Affiliations:** 1 Department of Dietetics, Krishna Institute of Medical Sciences, Secunderabad, IND; 2 Department of Food and Nutrition, Bhagini Nivedita College (BNC) University of Delhi, New Delhi, IND; 3 Department of Anatomy, All India Institutes of Medical Sciences (AIIMS) Vijaypur, Jammu, IND; 4 Department of Biochemistry, N. K. P. Salve Institute of Medical Sciences and Research Centre, Lata Mangeshkar Hospital, Nagpur, IND; 5 Department of Community Medicine, Ravindra Nath Tagore (RNT) Medical College, Udaipur, IND; 6 Department of Community Medicine, Dr. N. D. Desai Faculty of Medical Science and Research, Dharmsinh Desai University, Nadiad, IND; 7 Department of Homoeopathy, Vishva Advanced Homoeopathy Clinic, Pune, IND

**Keywords:** clinical nutrition, macronutrients, micronutrients, nutritional balance, precision nutrition

## Abstract

Nutrition plays a fundamental role in maintaining human health and modulating disease risk across the life course. This narrative review synthesizes contemporary evidence on the clinical significance of macronutrients, including carbohydrates, proteins, and fats, and micronutrients, including vitamins and minerals, establishing nutritional balance as a central determinant of human health, disease susceptibility, and therapeutic efficacy. These nutrient categories function within an integrated metabolic network in which macronutrients provide energy and structural substrates, while micronutrients serve as essential cofactors and regulatory agents in enzymatic, hormonal, and cellular signalling processes. The synthesis demonstrates that nutritional imbalance, arising from either deficiency, such as iron-deficiency anaemia and vitamin D insufficiency, or excess, including high intakes of refined carbohydrates and saturated fats, constitutes a major contributor to global disease burden, particularly the phenomenon described as the double burden of malnutrition. In response to these challenges, the review highlights the role of evidence-based nutritional therapy, encompassing established dietary patterns such as the Mediterranean and Dietary Approaches to Stop Hypertension (DASH) diets, as well as the clinical implementation of medical nutrition therapy in chronic disease management. It further emphasizes a paradigmatic shift from population-level dietary recommendations toward precision nutrition, an emerging framework that integrates nutrigenomics, metabolomics, and gut microbiome profiling to inform personalized dietary interventions. By conceptualizing nutrition as a dynamic and interactive system, this review offers a comprehensive perspective that integrates biochemical mechanisms with individualized clinical care, positioning nutritional balance as a foundational component of contemporary preventive and therapeutic medicine.

## Introduction and background

Nutrition is a fundamental determinant of human health, directly influencing multiple physiological processes, including cellular metabolism, immune regulation, and structural integrity across the lifespan [1]. Rather than serving merely as a dietary construct, it functions as a clinically actionable determinant of metabolic, immunological, and systemic homeostasis [1]. The human body operates as a dynamic and tightly regulated biochemical network in which macronutrients (carbohydrates, proteins, and fats) provide energy and structural substrates, while micronutrients (vitamins, minerals, and trace elements) serve primarily as enzymatic cofactors and modulators of cellular signaling pathways that contribute to gene regulation and homeostatic maintenance [2]. Coordinated interactions between these nutrient classes sustain growth, tissue repair, and disease modulation [3]. Nutritional balance should therefore be understood within a clinical and translational framework that extends beyond dietetics into preventive and therapeutic medicine [4].

Both nutrient deficiency and excess represent clinically measurable and epidemiologically significant conditions [5]. Adequate macronutrient intake supports energy metabolism, endocrine stability, and musculoskeletal integrity, whereas micronutrient sufficiency is required for neurocognitive function, immune competence, and antioxidant defense systems [6]. Disruptions in this balance initiate pathophysiological cascades rather than isolated deficiencies [7]. Protein deficiency impairs immune function and tissue repair, iron deficiency leads to anemia and cognitive impairment, and vitamin D deficiency disrupts calcium homeostasis and immune modulation [8]. Excessive consumption of refined carbohydrates and saturated fats is associated with obesity, type 2 diabetes, cardiovascular disease, and metabolic syndrome [9]. Nutritional imbalance, therefore, exists along a bidirectional spectrum of deficiency and excess with substantial global health implications [10].

Dietary patterns over recent decades have been shaped by globalization, urbanization, and industrialization, contributing to the double burden of malnutrition described by the World Health Organization (WHO) [11]. This phenomenon reflects the simultaneous presence of undernutrition and overweight within the same populations and continues to expand globally [12]. Countries historically affected by micronutrient deficiencies and growth stunting now face rising rates of obesity and non-communicable diseases driven by energy-dense, nutrient-poor dietary patterns, while developed nations contend with dietary excess and sedentary behavior. These trends underscore complex interactions between socioeconomic determinants, food systems, and health outcomes [13,14]. Nutrition, therefore, occupies a central role in global disease prevention and health promotion strategies [15].

Despite substantial advances in nutritional science, much of the literature has adopted a reductionist paradigm that isolates single nutrients rather than examining integrative dietary patterns and nutrient interactions [16]. Numerous studies detail the independent effects of specific compounds, such as vitamin C in immune modulation or omega-3 fatty acids in cardiovascular protection, and yet comparatively fewer analyses evaluate synergistic interactions between macronutrients and micronutrients within clinically relevant contexts [17]. Existing narrative and systematic reviews have largely emphasized isolated nutrient-disease associations, leaving a conceptual gap regarding integrative, clinically oriented synthesis that incorporates inter-nutrient dynamics and biological variability. Interindividual variability in nutrient metabolism, genetic polymorphisms, and gut microbiome composition further contributes to heterogeneous responses to comparable dietary exposures [18]. These factors challenge universal dietary recommendations and position precision nutrition as an emerging analytical framework [19]. Nutrigenomics, metabolomics, and microbiomics have advanced understanding of nutrient-gene and nutrient-microbiota interactions, enabling more individualized nutritional strategies [20].

Within this context, the transition from population-based dietary guidance to precision-oriented nutrition requires explicit conceptual integration of macronutrient and micronutrient balance across clinical and epidemiological domains. Nutrient requirements vary according to age, sex, genetic background, environmental exposures, and cultural dietary practices. Methodological limitations in dietary assessment and the effects of food processing further complicate the standardization of nutrient recommendations. These challenges define the translational boundary of current nutrition science and underscore the need for context-specific, evidence-based synthesis.

This review critically integrates clinical and epidemiological evidence on macronutrient-micronutrient interactions, with emphasis on nutritional balance, deficiency states, excess conditions, and therapeutic implications. Specifically, it addresses two central questions: (1) how do macronutrient-micronutrient interactions influence metabolic and clinical outcomes, and (2) what is the strength and limitation of current evidence across deficiency, excess, and therapeutic contexts? The objective is to delineate the clinical relevance, epidemiological scope, and translational implications of nutrient balance while identifying areas requiring further integrative investigation.

Methodology

This narrative review used a structured literature search designed to enhance transparency and comprehensiveness. The objective was to synthesize published evidence addressing the clinical implications of macronutrients and micronutrients, nutritional balance, deficiency states, excess conditions, and therapeutic applications.

Electronic databases, including PubMed, Scopus, and Web of Science, were systematically searched to identify relevant peer-reviewed literature. Google Scholar was used as a supplementary source to identify additional eligible records, with screening restricted to peer-reviewed journal articles. The search covered studies published between January 2015 and December 2024. The final literature search was completed in December 2024. The timeframe was selected to prioritize contemporary evidence reflecting current dietary patterns, evolving clinical practice, and advances in precision nutrition.

Search strategies combined controlled vocabulary terms and free-text keywords, including “macronutrients”, “micronutrients”, “clinical nutrition”, “nutrient deficiency”, “metabolic health”, “nutritional balance”, and “therapeutic interventions”, using Boolean operators (AND, OR). Reference lists of eligible articles were manually screened to identify additional relevant publications. Titles and abstracts retrieved through database searches were screened for relevance, followed by full-text evaluation of potentially eligible articles. Study selection was based on predefined inclusion and exclusion criteria aligned with the objectives of the review.

Eligibility criteria included peer-reviewed human studies with direct clinical or physiological relevance. Studies were included if they examined nutrient exposure, dietary patterns, supplementation, or deficiency states in human populations and reported clinical, metabolic, or epidemiological outcomes. Study designs considered eligible comprised randomized controlled trials, clinical trials, observational cohort and case-control studies, systematic reviews, and meta-analyses. Animal studies, non-clinical experimental studies without clear translational relevance, conference abstracts, commentaries, and grey literature were excluded.

Given the narrative design, no formal risk-of-bias assessment or quantitative meta-analysis was performed. Evidence was synthesized descriptively, with attention to consistency of findings across study designs and relevance to clinical practice. When conflicting evidence was identified, greater interpretive weight was assigned to higher levels of evidence, particularly meta-analyses and randomized controlled trials. Key variables extracted from eligible studies included study design, population characteristics, nutrient exposure or intervention, primary outcomes, and principal findings relevant to clinical interpretation.

Evidence interpretation followed a study-design hierarchy. Randomized controlled trials and meta-analyses were regarded as higher level interventional evidence; observational studies were interpreted as associative evidence; and mechanistic or experimental findings were considered hypothesis-generating. Distinctions between association and causation were explicitly acknowledged where applicable.

This methodological approach was intended to provide an integrative and clinically oriented synthesis rather than a systematic review conducted under PRISMA guidelines. Accordingly, the manuscript is presented as a structured narrative review.

## Review

Macronutrients: the foundation of metabolic health

Integrated Physiological Roles

Carbohydrates, proteins, and fats collectively sustain energy metabolism, structural integrity, and physiological regulation; however, they operate within an integrated and tightly regulated metabolic system rather than as independent determinants of metabolic outcomes [21]. In addition to functioning as substrates, these macronutrients participate in signalling pathways that interact with endocrine and immune systems, although overall metabolic regulation remains multifactorial and influenced by genetics, total energy intake, physical activity, environmental exposures, and neuroendocrine regulation [22]. Evidence supporting these roles derives primarily from mechanistic and physiological studies, whereas clinical outcome modification depends on broader dietary patterns and long-term energy balance.

Macronutrient Proportions and Metabolic Flexibility

The relative proportion and quality of macronutrients contribute to metabolic responses, but current evidence indicates that these effects are context-dependent rather than universally prescriptive [3]. Carbohydrates function as primary oxidative substrates through glycolysis and the tricarboxylic acid cycle, proteins provide amino acids necessary for enzymatic activity and tissue turnover, and lipids contribute to membrane structure and bioactive mediator synthesis [23,24]. These functions are well established at the biochemical level; however, translation to clinical outcomes varies according to study design and population characteristics.

These physiological roles occur within the framework of metabolic flexibility, defined as the capacity to adapt substrate utilization according to energetic demand and hormonal signalling [25]. Impairment of metabolic flexibility has been observed in insulin-resistant and obese phenotypes; however, whether macronutrient redistribution alone restores this flexibility remains inconsistently demonstrated in long-term randomized trials. Most supportive data originate from short-term metabolic ward studies rather than extended clinical endpoint trials.

Energy Balance Versus Macronutrient Ratios

Dietary patterns characterized by high intakes of refined carbohydrates and saturated fats are consistently associated in epidemiological studies with obesity, insulin resistance, and cardiovascular disease; however, these relationships are mediated not only by macronutrient composition but also by sustained caloric excess and long-term energy imbalance [9]. Such associations should not be interpreted as direct causation, as residual confounding and lifestyle covariates frequently influence observational findings.

Ongoing scientific debate persists regarding whether metabolic outcomes are primarily driven by macronutrient ratios or by overall energy balance. Interventional evidence indicates that energy balance remains central to weight regulation, whereas macronutrient distribution may influence satiety, insulin dynamics, and adherence in specific clinical contexts [5]. Comparative trials evaluating low-carbohydrate versus balanced dietary patterns frequently demonstrate similar weight loss and cardiometabolic improvements when caloric intake is equivalent, suggesting that macronutrient composition modulates but does not override energy balance. This pattern reinforces the primacy of caloric balance in randomized settings.

Population Guidance and Individual Variability

Standard macronutrient distribution ranges (e.g., 50%-60% carbohydrates, 20%-30% fats, 10%-20% proteins) serve as general population guidance but should not be interpreted as universally optimal targets [8]. Macronutrient requirements vary according to age, metabolic phenotype, disease state, and activity level. For example, lower carbohydrate dietary patterns may improve glycemic control in selected individuals with insulin resistance, whereas balanced dietary approaches demonstrate comparable long-term cardiovascular outcomes in broader populations. Effect sizes in glycemic improvement trials are generally moderate and often attenuate over extended follow-up periods, underscoring the importance of adherence and baseline metabolic status. Therefore, evidence supporting specific macronutrient prescriptions is stronger for short-term metabolic markers than for sustained hard clinical endpoints. These findings underscore the absence of a single superior macronutrient ratio.

Metabolic Synergy and Mechanistic Interpretation

The concept of “metabolic synergy” refers to interactive physiological effects among macronutrients. Experimental and short-term interventional studies suggest that protein co-ingestion may attenuate postprandial glycemic excursions, unsaturated fats may enhance insulin sensitivity, and dietary fibre may influence lipid metabolism [12]. However, the magnitude and durability of these effects vary across trials, and long-term clinical endpoints are not consistently demonstrated. These interactions should therefore be interpreted as modulatory rather than determinative mechanisms.

Mechanistic findings describing substrate competition, incretin modulation, or lipid-mediated signalling pathways should be regarded as biologically plausible frameworks rather than definitive evidence of clinical outcome modification. Such mechanisms provide explanatory context but do not substitute for randomized outcome data.

Evidence Hierarchy and Clinical Interpretation

Clear differentiation between associative and interventional evidence is required. Observational studies consistently link certain dietary patterns to reduced cardiometabolic risk, whereas randomized controlled trials demonstrate that improvements are often contingent upon energy control, dietary adherence, and baseline metabolic phenotype [10]. The evidence further indicates heterogeneity in outcomes across study designs, reinforcing the need to interpret associations cautiously and avoid extrapolating causality from non-interventional data [9]. Accordingly, macronutrient balance should be conceptualized as adaptive and individualized rather than static. Table 1 summarizes these functional roles within a context-dependent metabolic framework rather than implying fixed macronutrient prescriptions.

**Table 1:** Summary of Macronutrient Functions, Metabolic Roles, and Clinical Implications

Macronutrient	Primary Physiological Roles	Key Metabolic Pathways	Clinical Implications of Imbalance	References
Carbohydrates	Primary energy source; maintain glucose homeostasis; support neural and muscular function	Glycolysis and the tricarboxylic acid (TCA) cycle are key pathways that interact with proteins and fats to sustain metabolic balance	Excess refined carbohydrates are linked with obesity, insulin resistance, and type 2 diabetes	[19,21,23]
Proteins	Provide structural integrity (muscles, enzymes, hormones); regulate immune response and tissue repair	Amino acid signalling and enzymatic regulation; reduce glycemic peaks when balanced with carbohydrates	Deficiency causes sarcopenia and impaired wound healing; excess protein stresses renal metabolism	[21,23,25]
Fats (lipids)	Provide energy density; essential for cell membrane integrity and hormonal regulation	Beta-oxidation; lipid signalling cascades; omega-6/omega-3 ratio critical for inflammation control	Excess saturated/trans fats promote atherosclerosis; unsaturated fats improve cardiovascular and metabolic health	[19,20,21]
Overall nutrient balance	Dynamic equilibrium ensures metabolic flexibility and long-term health	Ideal distribution: 50–60% carbohydrates, 20–30% fats, 10–20% proteins; varies by age and health status	Imbalance leads to obesity, insulin resistance, and metabolic disorders	[1,21,25]

Carbohydrates and glycemic control

Structural Characteristics and Postprandial Physiology

Carbohydrates remain a principal energy source in human metabolism, although their physiological effects are influenced by molecular structure, digestibility, food matrix context, and co-ingested macronutrients [26]. The absorption of simple sugars such as glucose, fructose, and sucrose produces relatively rapid postprandial elevations in blood glucose and insulin concentrations [27]. In contrast, complex carbohydrates and dietary fibre are generally associated with slower glycemic responses and more gradual insulin secretion [28].

These postprandial dynamics are commonly quantified using the glycemic index (GI) and glycemic load (GL); however, their predictive value for long-term glycemic control and clinical outcomes remains variable across populations and dietary contexts [9]. GI and GL values are derived under standardized single-food testing conditions and may not fully account for mixed-meal composition, inter-individual glycemic variability, food processing, or metabolic phenotype.

High-GI Diets and Metabolic Risk

In epidemiological studies, high-GI dietary patterns have been associated with hyperinsulinaemia, oxidative stress, and inflammatory markers, processes implicated in insulin resistance and type 2 diabetes mellitus (T2DM) pathogenesis [29]. Causal inference, however, is limited by confounding dietary and lifestyle variables, and randomized controlled trials have shown heterogeneous results regarding long-term diabetes prevention.

Persistent hyperglycemia contributes to β-cell dysfunction and impaired insulin signalling, mechanisms central to metabolic syndrome development [10]. These mechanistic pathways are well characterized experimentally, although the extent to which GI reduction alone prevents progression to T2DM remains debated. Furthermore, glycemic responses differ substantially according to baseline insulin sensitivity, physical activity level, adiposity, and genetic background, limiting the uniform application of GI-based dietary prescriptions.

Dietary Fibre and Glycemic Modulation

In observational studies and selected intervention trials, low-GI foods such as whole grains, legumes, and vegetables have been associated with improved glycemic control, lipid profiles, and satiety [27]. Clinical data from the 2015-2025 period indicate reductions in fasting glucose, glycated hemoglobin (HbA1c), and triglycerides with low-GI dietary patterns; however, effect sizes are generally modest and frequently attenuated over longer follow-up periods, particularly when caloric intake is not concurrently controlled [30].

Fibre intake is similarly associated with improved GIs [23]. Soluble fibres delay gastric emptying and glucose absorption, whereas insoluble fibres influence intestinal transit and postprandial variability [25]. Evidence supporting these effects derives largely from short- to medium-term interventional studies, with stronger consistency for metabolic markers than for hard cardiovascular endpoints. Response to increased fibre intake varies across individuals, and gastrointestinal tolerance, habitual intake, and microbiome composition influence both adherence and metabolic benefit.

The Intestinal-Metabolic Axis

Beyond mechanical effects, fibre modulates gut microbiota composition and promotes short-chain fatty acid production, which has been linked mechanistically to improved insulin sensitivity and immunometabolic regulation [18]. This intestinal-metabolic axis provides a plausible biological framework connecting carbohydrate quality to systemic outcomes [22]. Nonetheless, most microbiome-related findings remain mechanistic or hypothesis-generating, with limited long-term randomized evidence demonstrating sustained clinical benefit. Translation of microbiome modulation into reproducible clinical endpoints remains an area of ongoing investigation rather than an established therapeutic consensus.

Fructose, Added Sugars, and NAFLD Risk

Excess fructose intake, particularly from sugar-sweetened beverages, has been associated with hepatic lipogenesis, hyperuricemia, and ectopic fat accumulation, contributing to non-alcoholic fatty liver disease (NAFLD) risk [24,26]. These associations are strongest in the context of caloric surplus, and controlled isocaloric substitution trials suggest that fructose-related harm is amplified when excess energy intake is present. Distinguishing intrinsic sugars within whole fruits from added sugars in processed foods is therefore clinically relevant for dietary guidance [28]. Whole-fruit consumption, despite intrinsic sugar content, is not consistently associated with adverse metabolic outcomes, likely due to fibre content, food matrix effects, and portion size modulation.

Quantity, Quality, and Precision Approaches

Evidence from the past decade indicates that both carbohydrate quantity and quality influence metabolic outcomes; however, comparative trials suggest that total energy balance often exerts a stronger effect on weight and cardiometabolic endpoints than carbohydrate restriction alone [12]. Emphasis on complex carbohydrates, high fibre intake, and limitation of added sugars aligns with contemporary clinical nutrition guidance [8].

Rather than replacing conventional dietary advice, emerging technologies, such as continuous glucose monitoring and machine-learning-assisted glycemic prediction models, aim to refine carbohydrate recommendations by accounting for individual variability in glycemic responses. Although these technologies enable individualized glycemic profiling, their long-term clinical utility and cost-effectiveness in routine metabolic care remain under evaluation [30].

Proteins

Structural and Functional Roles of Proteins

Proteins are essential macronutrients that contribute to structural integrity and regulatory processes [31]. They consist of 20 amino acids, nine of which are classified as essential, and support nitrogen balance, enzyme and hormone synthesis, and immune function [32]. These physiological roles are well established; however, clinical outcomes associated with protein intake vary according to population characteristics, baseline nutritional status, and disease context.

Protein quality is determined by amino acid composition and bioavailability, influencing its capacity to support lean body mass and cellular function [8]. Proteins of animal origin generally provide complete amino acid profiles, whereas plant proteins may require complementary combinations to achieve comparable amino acid adequacy [5]. Nevertheless, well-planned plant-based diets can meet essential amino acid requirements without animal sources, and long-term health outcomes appear more strongly influenced by overall dietary patterns than by amino acid completeness alone. Beyond structural functions, amino acids participate in neurotransmission, intracellular signalling, and gene regulation [33]. Protein turnover, reflecting the balance between synthesis and degradation, is central to tissue maintenance and metabolic adaptation [12].

Protein in Catabolic States and Clinical Recovery

Protein catabolism increases in hypermetabolic and inflammatory states such as infection, trauma, and surgery; adequate protein intake during these conditions is associated with improved nitrogen balance and wound healing [34]. Evidence supporting improved clinical outcomes derives primarily from randomized controlled trials in hospitalized, post-surgical, critically ill, or malnourished populations. In these groups, outcomes such as preservation of lean body mass, improved functional recovery, reduced complications, and shorter rehabilitation duration have been reported, although effect sizes are generally modest and influenced by baseline nutritional status.

Recent data from 2015-2025 suggest that optimized protein intake may improve rehabilitation metrics in older adults with sarcopenia and in selected clinical populations [31]. However, heterogeneity in dosing strategies, timing of administration, and patient phenotype limits universal prescription, and evidence in well-nourished, community-dwelling adults remains less consistent.

Protein Requirements in Ageing and Sarcopenia

In older adults, inadequate protein intake is associated with sarcopenia, characterized by progressive loss of muscle mass and strength [35]. An intake of approximately 1.0-1.2 g/kg body weight/day is commonly recommended to support muscle protein synthesis, particularly when distributed evenly across meals [9]. Randomized trials indicate modest improvements in lean mass and functional performance when adequate protein intake is combined with resistance training.

The anabolic response to protein intake is mediated in part through activation of the mechanistic target of rapamycin (mTOR) pathway, stimulated by essential amino acids such as leucine and potentiated by resistance exercise [27]. Most evidence supporting mTOR activation derives from acute metabolic and feeding studies, and long-term functional outcomes depend on physical activity, energy balance, and anabolic sensitivity. Chronic overstimulation of mTOR signalling has been hypothesized to contribute to ageing-related pathways and certain oncogenic processes; however, clinical evidence linking dietary leucine intake within recommended ranges to adverse outcomes remains inconclusive. Thus, the interaction between protein intake and mTOR activity should be interpreted within physiological intake ranges rather than pharmacologic extremes.

Protein Intake in Chronic Disease Contexts

Protein requirements vary in chronic disease states [18]. In chronic kidney disease (CKD), excessive protein intake may exacerbate hyperfiltration, whereas inadequate intake increases the risk of protein-energy wasting; therefore, stage-specific dietary prescriptions are recommended [26]. Randomized trials in individuals without CKD do not consistently demonstrate clinically significant renal impairment with higher protein intakes within typical dietary ranges, indicating that renal risk is population-specific rather than universal.

In hepatic dysfunction, protein quantity and type influence ammonia metabolism and encephalopathy risk [24]. Specialized formulations, including branched-chain amino acid supplementation, have demonstrated benefit in selected cirrhotic populations [32]. These benefits are primarily documented in moderate-to-severe disease and should not be extrapolated beyond hepatic impairment.

Protein, Weight Regulation, and Cardiometabolic Risk

Higher protein diets are associated with increased satiety, greater thermic effect of food, and modulation of appetite-regulating hormones such as ghrelin and peptide YY [34]. Short- to medium-term randomized trials demonstrate modest improvements in weight loss and body composition when protein replaces refined carbohydrates under controlled caloric conditions; however, long-term superiority over balanced dietary patterns remains inconsistent, particularly when total energy intake is comparable.

The source of protein is also relevant [35]. Observational studies associate higher intake of plant-derived proteins with reduced cardiometabolic risk and lower incidence of certain cancers, whereas greater consumption of red and processed meats correlates with increased chronic disease risk [12]. These associations are influenced by overall dietary patterns, substitution effects, and lifestyle factors, limiting direct causal inference. Evidence regarding oncologic outcomes remains heterogeneous across cancer types and study designs, and protective effects appear more consistent when plant proteins replace processed meats within broader high-quality dietary patterns.

Sustainability and Precision Approaches

Dietary models such as the EAT-Lancet Planetary Health Diet emphasize predominantly plant-based protein patterns to align nutritional adequacy with environmental sustainability [10]. While such models integrate ecological considerations, their clinical superiority for specific metabolic or disease endpoints remains under evaluation.

**Figure 1:**
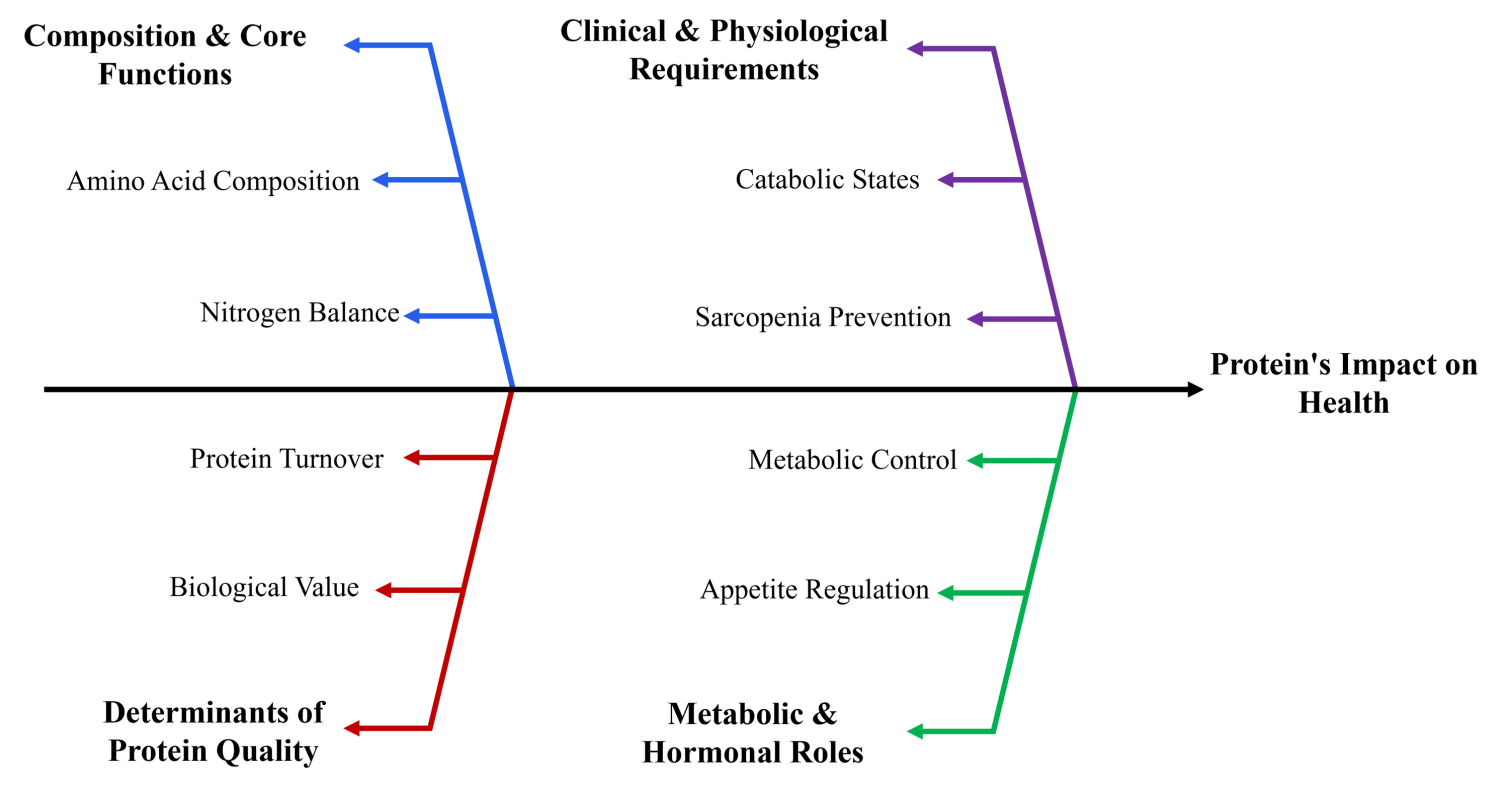
Protein’s multifaceted role in health Created by authors using Microsoft PowerPoint (Microsoft Corporation, Redmond, USA)

Overall, proteins contribute to structural maintenance, metabolic regulation, and recovery processes [31]. Sufficient, high-quality, and context-specific protein intake supports cellular and systemic resilience [33]. Future precision-nutrition approaches aim to tailor protein recommendations according to age, metabolic phenotype, comorbidity profile, and physical activity patterns rather than applying uniform intake targets. Figure 1 illustrates that protein-related health effects depend on quantity, quality, metabolic context, and individual variability.

Fatty acids and fats in cardiac metabolism

Dietary Fats and Cardiometabolic Regulation

Dietary fats are essential components of human physiology, contributing to energy provision, membrane structure, and metabolic regulation [35,36]. Fatty acids also function as bioactive molecules that influence inflammatory signaling, vascular tone, and lipid transport, all of which are relevant to cardiometabolic health [37]. The physiological effects of dietary fats depend not only on quantity but also on fatty acid composition, food source, and dietary context [5].

Linoleic acid (omega-6) and alpha-linolenic acid (omega-3) are essential fatty acids that must be obtained from the diet [38]. Through elongation and desaturation pathways, these precursors give rise to longer chain derivatives, including arachidonic acid, eicosapentaenoic acid (EPA), and docosahexaenoic acid (DHA), which participate in eicosanoid and specialized pro-resolving mediator synthesis [12]. Omega-6-derived metabolites may contribute to pro-inflammatory signaling under certain conditions, whereas omega-3-derived mediators are involved in inflammation resolution pathways [39]. However, these pathways are tightly regulated, and inflammatory responses depend on overall dietary pattern, metabolic status, and immune activation rather than fatty acid class alone [40].

Omega-6:Omega-3 Ratio - Interpretation and Controversy

Omega-3 fatty acids have been investigated for cardiometabolic benefits, including triglyceride reduction, endothelial function improvement, and modulation of inflammatory pathways [41]. Dose-dependent reductions in triglyceride concentrations are consistently demonstrated in randomized trials, particularly at higher pharmacologic doses. However, evidence regarding the decrease in major cardiovascular events remains heterogeneous. While some large-scale trials have reported benefit in selected high-risk populations, other contemporary randomized controlled trials have demonstrated neutral effects on hard cardiovascular endpoints [42]. These inconsistencies suggest that clinical benefit may depend on formulation, dosage, baseline cardiovascular risk, and background dietary patterns rather than representing a uniform cardioprotective effect. Mechanistic studies support anti-inflammatory actions mediated through lipid signaling and nuclear factor-κB modulation [27]; however, translation of these mechanisms into consistent clinical outcomes (i.e., reduction in disease progression) remains context-specific.

Lipid Metabolism and Lipoprotein Transport

Dietary triglycerides are absorbed, hydrolysed, re-esterified, and packaged into chylomicrons for systemic distribution; lipoprotein lipase facilitates fatty acid uptake into tissues for oxidation or storage [23]. Lipid metabolism is highly regulated and influenced by genetic polymorphisms, insulin sensitivity, and baseline dietary composition [18]. Inter-individual variation in desaturase enzyme activity further modifies fatty acid conversion efficiency and metabolic outcomes [25].

Saturated Fats, Substitution Effects, and Cardiovascular Risk

Higher intake of saturated and trans fats has been associated with increased low-density lipoprotein cholesterol and elevated cardiovascular risk in observational studies [40]. However, clinical interpretation requires consideration of substitution effects. Randomized trials and pooled analyses indicate that replacing saturated fats with polyunsaturated fats is associated with improved lipid profiles and reduced cardiovascular events, whereas replacement with refined carbohydrates does not confer similar benefits. Furthermore, cardiovascular risk may vary according to food source, as saturated fats derived from processed meats may not exert effects identical to those from dairy products. Thus, saturated fats should not be viewed as uniformly harmful independent of dietary context.

Dietary patterns rich in unsaturated fats, such as the Mediterranean diet, have demonstrated reductions in coronary morbidity and mortality in large randomized and observational studies [30]. These benefits likely reflect composite dietary patterns rather than isolated fatty acid effects.

Omega-3 Fatty Acids and Cardiovascular Outcomes

Omega-3 fatty acids influence endothelial function, platelet aggregation, and hepatic triglyceride synthesis [41]. Mechanistically, EPA and DHA participate in the modulation of inflammatory transcription pathways, including nuclear factor-κB signaling [27]. Robust evidence supports dose-dependent reductions in circulating triglyceride levels, particularly at pharmacologic doses. However, evidence for secondary cardioprotection remains heterogeneous. While certain large-scale trials have reported reductions in cardiovascular events in high-risk populations receiving purified EPA formulations, other contemporary randomized studies have demonstrated neutral effects on major adverse cardiovascular endpoints. Therefore, omega-3 supplementation appears most consistently beneficial for triglyceride lowering and may confer cardiovascular benefit in selected high-risk populations rather than universally across all groups.

Clinical Implications and Dietary Context

Dietary fats should be evaluated according to their molecular composition, food matrix, and substitution patterns rather than total quantity alone [10]. Replacement of saturated and trans fats with mono- and polyunsaturated fats remains a central strategy in cardiovascular risk reduction guidelines [36]. At the same time, heterogeneity in baseline diet, metabolic phenotype, and genetic variation influences individual response to fat modification.

Overall, the cardiometabolic effects of dietary fats are context-dependent, and simplified causal models linking individual fatty acid classes directly to disease outcomes fail to capture the complexity demonstrated in contemporary clinical evidence.

Vitamins: the catalysts of cellular function

Vitamins are essential micronutrients required for enzymatic catalysis, redox balance, and cellular signalling [2]. Although required in small quantities, both deficiency and excess can disrupt metabolic homeostasis and cause clinically recognizable disease states [43]. Vitamins are classified as fat-soluble (A, D, E, K) or water-soluble (B-complex, C) according to their solubility and storage properties [5].

Vitamin A regulates epithelial differentiation, visual function, and immune competence; its deficiency leads to xerophthalmia and impaired immunity, whereas excess intake may result in hepatotoxicity and teratogenicity [8]. Vitamin D, synthesized cutaneously and activated to calcitriol, plays a central role in calcium-phosphate homeostasis and skeletal integrity [44]. Beyond skeletal function, vitamin D has been associated with modulation of immune and endocrine pathways; however, evidence for non-skeletal outcomes is largely associative or context-dependent, and randomized trials have yielded mixed findings outside deficiency states. Vitamin E functions as a lipid-phase antioxidant that limits membrane lipid peroxidation, and vitamin K is required for γ-carboxylation of coagulation factors and bone-related proteins [45]. Emerging data suggest potential interactions between vitamins D and K in skeletal and vascular biology, although clinical implications of combined supplementation remain under investigation [46].

Water-soluble vitamins primarily act as coenzymes in intermediary metabolism [9]. The B-complex group (thiamine, riboflavin, niacin, pyridoxine, folate, and cobalamin) supports carbohydrate oxidation, neurotransmitter synthesis, and DNA methylation processes [18]. Deficiencies result in well-characterized syndromes such as beriberi, pellagra, and megaloblastic anemia [10]. Vitamin C contributes to collagen synthesis, iron absorption, and antioxidant defense mechanisms [27].

Vitamin Supplementation: Corrective Versus Preventive Use

Vitamin supplementation is clinically corrective in confirmed deficiency states and remains standard care in such contexts [25]. In contrast, evidence supporting supplementation in non-deficient populations is variable and often inconsistent across micronutrients. For example, folate and vitamin B12 supplementation reduces homocysteine concentrations; however, reductions in cardiovascular events have not been uniformly demonstrated in randomized trials [43]. Vitamin D supplementation consistently improves bone mineral density in deficient individuals, whereas benefits for immune modulation and endocrine function, or cardiometabolic outcomes remain population-specific and not uniformly established.

Indiscriminate supplementation may lead to hypervitaminosis or nutrient imbalance, particularly for fat-soluble vitamins [44]. Consequently, clinical guidelines generally recommend targeted supplementation based on deficiency risk, laboratory confirmation, or defined clinical indications rather than routine high-dose use in the general population.

Food-First Approaches and Precision Micronutrition

**Figure 2:**
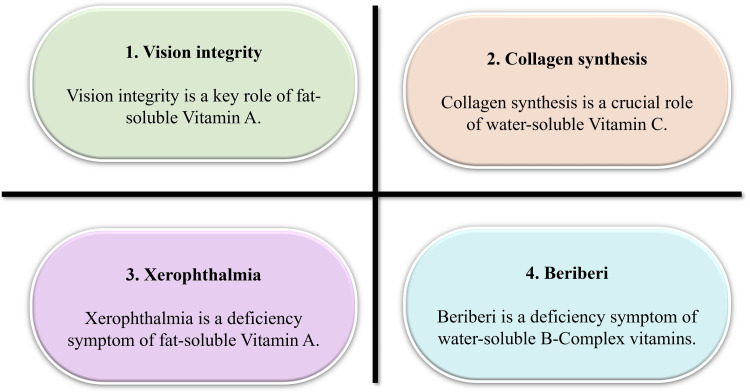
Key physiological roles and deficiency symptoms of fat- and water-soluble vitamins Created by authors using Microsoft PowerPoint (Microsoft Corporation, Redmond, USA)

A food-first strategy, emphasizing whole-food dietary sources over pharmacologic dosing, is widely supported in preventive nutrition frameworks [45]. Whole-food matrices provide synergistic nutrient interactions that are not fully replicated by isolated supplements. Advances in nutrigenomics suggest that genetic polymorphisms influence vitamin metabolism, transport, and activation pathways [46]. These findings support exploration of personalized micronutrient strategies; however, clinical application remains in development, and robust outcome-based trials are still limited. Figure 2 illustrates the physiological roles of selected vitamins and the clinical manifestations associated with deficiency states.

Minerals and trace elements in physiological homeostasis

Trace elements and minerals maintain enzymatic activity, electrochemical gradients, and structural stability within the organ systems [47]. They are essential in the regulation of cardiovascular, neurological, and skeletal health [48]. Iron plays a major role in the transportation of oxygen and in mitochondrial respiration [12]. Its deficiency induces anemia and cognitive impairment, and overload causes oxidative stress and hepatic fibrosis [49]. Calcium is the primary mineral in bones, and its imbalance results in osteopenia or vascular calcification [50]. Depending on vitamin D status, it is interdependent on the micronutrients [44].

Magnesium is a cofactor of over 300 enzymatic reactions associated with the production of ATP and the metabolism of nucleic acids [8]. It has been shown that hypertension, insulin resistance, and arrhythmia are associated with poor magnesium consumption, whereas in good amounts, it promotes endothelial and metabolic activity [27]. Zinc aids in DNA synthesis, immune response, and tissue healing; its deficiency hinders growth and immunity, and excess prevents copper uptake [51]. Examples of trace elements with endocrine effects are selenium and iodine [42]. Selenium, which is a component of a series of selenoproteins, e.g., glutathione peroxidase, helps to counteract oxidative damage and affects thyroxine and triiodothyronine production [45]. Its deficiency as well as excess may upset thyroid and cardiovascular homeostasis [46]. Universal salt iodization has significantly decreased the deficiency disorders, but needs to be monitored to prevent overexposure [47].

Current studies emphasize extensive interactions between micronutrients that jointly influence the physiological outcomes [48]. Magnesium regulates the action of vitamin D, zinc and copper compete in the absorption, and selenium affects the efficiency of the thyroid [49]. These interactions expose the shortcomings of individual nutrient supplementation and embrace whole-diet interventions based on vegetables, legumes, lean proteins, and fortified grains [50]. New data indicate that mineral status, too, is connected with epigenetic regulation, and trace elements alter gene-expression patterns that may be related to both metabolic and neurodegenerative diseases [51]. Basically, minerals and trace elements act as noiseless controllers of the systemic balance [52]. Enzymatic efficiency, immune competence, and neurological stability are maintained by keeping micronutrients in their optimal levels using a balanced diet and evidence-based supplementation [50]. Modern clinical nutrition is now adopting a systems-based perspective that sees the micronutrients as interacting components of a single metabolic system [52].

Nutrient deficiency disorders: global epidemiology and clinical manifestations

Nutrient deficiencies have a significant impact on global health and contribute to high morbidity, mortality, and developmental handicapping [32]. Although there is significant improvement in food technology and healthcare systems, there are still broad gaps in both macro- and micronutrient intake due to bad diets, malabsorption, and socioeconomic inequalities [18]. The last 10 years (2015-2025) have witnessed a growing awareness of the so-called hidden hunger, a subclinical malnutrition of micronutrients that exists alongside an adequate caloric intake, especially in low- and middle-income groups [27]. Iron deficiency anemia (IDA) is the most common nutritional disorder in the world, with approximately one-third of the world population affected by the condition [35]. Since iron is required in the production of hemoglobin and transportation of oxygen, a shortage causes fatigue, loss of brain functions, and immunological dysfunction [13]. Children are particularly susceptible, as are women of reproductive age [26]. Oral supplementation and fortification of food have decreased the prevalence of anemia, but side effects such as gastrointestinal intolerance lead to poor compliance [19]. New developments like encapsulated iron and the biofortified crops (iron-rich rice and beans) are enhancing iron bioavailability and reducing such side effects [23]. The problem of vitamin D deficiency has been globally proportionate as it has afflicted both warm and cool climate areas, driven by factors such as lack of sun exposure, skin pigmentation, and a sedentary lifestyle [37]. In addition to the skeletal functions, vitamin D controls immune, endocrine, and metabolic functions [25]. Osteoporosis, autoimmune diseases, and cardiometabolic disorders are associated with low serum levels of 25-hydroxyvitamin D [12]. Research conducted between 2015 and 2025 identifies fortification, supplementation, and education of the population to mitigate this universal deficiency [38]. The most commonly preventable cause of cognitive impairment is still iodine deficiency [20]. Even though global salt iodization has decreased the prevalence of endemic goitre, inconsistent adoption and the reputation of speciality salts containing no iodide pose a threat to the long-term improvement [28]. Even mild deficiency in pregnant ladies may severely affect the neurodevelopment of the fetus, and this explains why regular intake of iodine during pregnancy is essential [15].

The other clinically important deficiencies are those of zinc, which causes immune suppression and slow wound healing [40]; vitamin A, which causes xerophthalmia and predisposes one to infection [24]; and vitamin B12 and folate, which cause megaloblastic anemia and neural-tube defects [29]. The lack of calcium and magnesium promotes osteoporosis and hypertension [17], whereas the lack of selenium is associated with cardiomyopathy and thyroid conditions [21]. Such circumstances prove that malnutrition goes beyond the lack of energy to subtle biochemical disequilibrium having systemic implications [16]. Their tenacity despite food abundance enhances the necessity to dietetically diversify, fortify, and do context-sensitive public-health interventions [30]. Table 2 shows that nutrient deficiencies remain major global health concerns, affecting diverse populations and requiring targeted interventions for prevention and control.

**Table 2:** Global Overview of Major Nutrient Deficiency Disorders

Nutrient Deficiency	Major Clinical Manifestations	Vulnerable Populations	Public Health/Clinical Interventions	References
Iron (iron deficiency anemia)	Fatigue, anemia, cognitive dysfunction, and immune impairment	Children, women of reproductive age	Food fortification, oral and encapsulated iron supplements, and biofortified crops	[13,17]
Vitamin D	Osteoporosis, autoimmune and cardiometabolic disorders	All age groups, sedentary individuals	Fortification, supplementation, and lifestyle changes, exposure to sunlight	[21]
Iodine	Goitre, hypothyroidism, impaired fetal neurodevelopment	Pregnant women, low-iodine regions	Universal salt iodization, prenatal supplementation	[15]
Zinc	Impaired immunity, delayed wound healing, and growth retardation	Low- and middle-income populations	Dietary diversification, zinc supplementation	[40]
Vitamin A	Xerophthalmia, increased infection risk	Children, undernourished populations	Vitamin A supplementation, food fortification	[24]
Vitamin B12 and folate	Megaloblastic anemia, neural tube defects	Pregnant women, the elderly, vegetarians	Combined folate and B12 supplementation, fortified grains	[29]
Calcium and magnesium	Osteoporosis, hypertension, and neuromuscular dysfunction	Elderly, postmenopausal women	Dietary fortification, supplementation, balanced intake	[32]
Selenium	Cardiomyopathy, thyroid dysfunction	Regions with selenium-deficient soil	Balanced diet, controlled supplementation	[37]

Nutritional toxicity and excess: the two-edged sword

Excessive intake of nutrients is equally problematic as the lack, and is an aspect of the worldwide nutrition transition: processed food, refined carbohydrates, and saturated fat are consumed in great amounts [28]. Excessive nutritional supplementation, mistakes in industrial food fortification, and uncontrolled excess of macronutrients can cause nutritional toxicity that can lead to a destabilization of metabolic homeostasis [41]. Hypervitaminosis is caused by excessive intake of micronutrients [17]. Fat-soluble vitamins (A, D, E, K) are stored in the body and can be toxic with consistent large amounts of intake [44]. Excess vitamin A results in hepatic damage and teratogenicity; excess vitamin D results in hypercalcemia and nephrocalcinosis; excess vitamin E results in increased risk of hemorrhage; and excess vitamin K interferes with anticoagulant treatment [33]. Such risks are increasing with the increase in the use of unmonitored supplements, and hence, medical supervision and evidence-based dosing are of great importance [36]. Iron overload is one of the examples of mineral toxicity caused by oxidative damage [14]. The accumulation of iron in the liver, pancreas, and heart, leading to fibrosis, diabetes, and cardiomyopathy, is found in hereditary hemochromatosis or with repeated blood transfusions [46]. Excess selenium (selenosis) causes gastrointestinal disturbance and neuropathy, whereas large amounts of zinc result in copper absorption impairment and immune impairments [29]. Water-soluble vitamins are also toxic at supraphysiologic doses - excess niacin can induce hepatotoxicity, and excess pyridoxine may initiate neuropathy [22].

**Figure 3:**
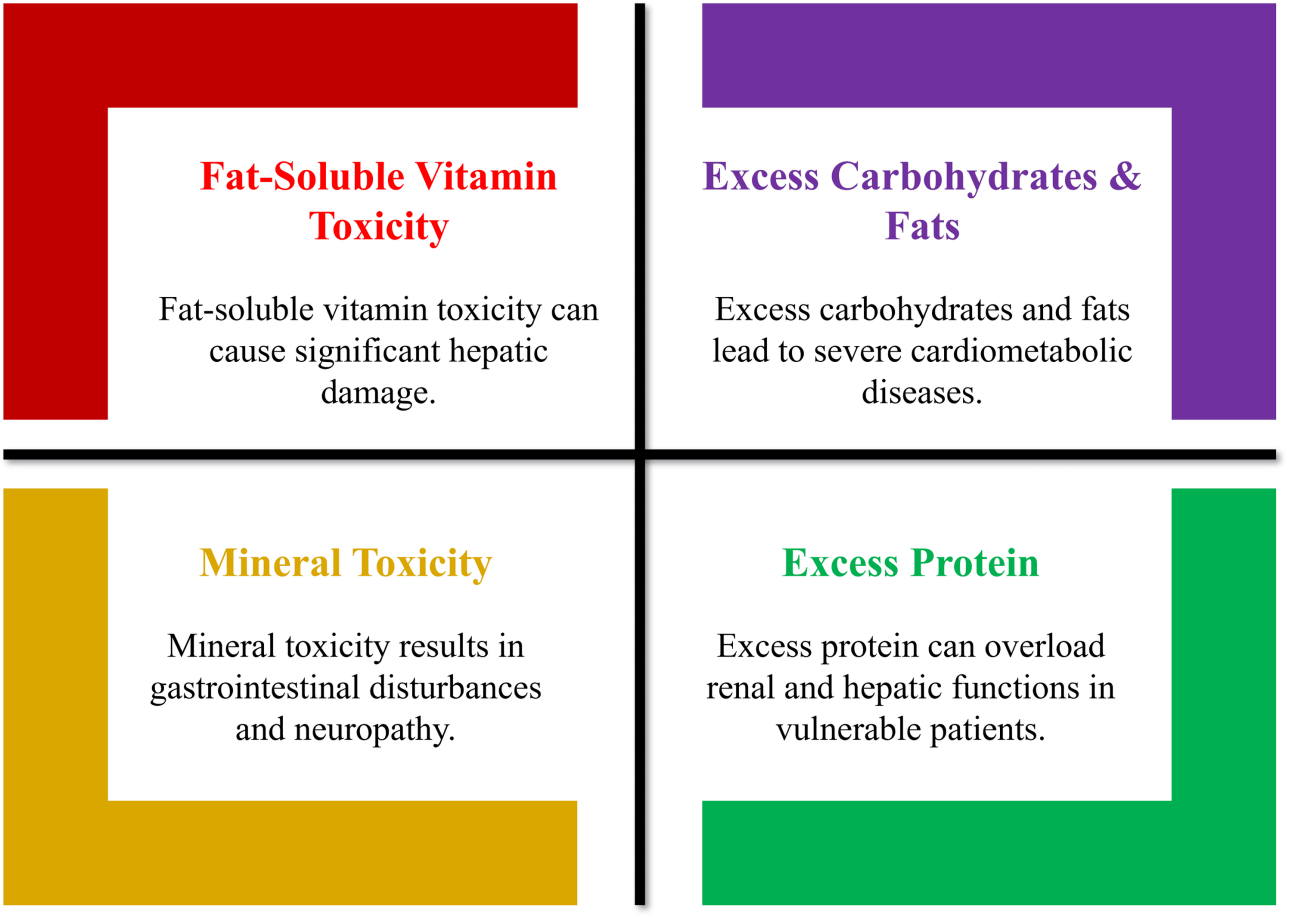
Health risks associated with excessive nutrient intake Created by authors using Microsoft PowerPoint (Microsoft Corporation, Redmond, USA)

Overconsumption of macronutrients is the most common form of nutrient imbalance [19]. High-carbohydrate and high-saturated fat diets induce insulin resistance, dyslipidemia, and NAFLD, and chronic caloric excess may result in obesity, the primary cause of cardiometabolic disorders [47]. Protein toxicity is not common, but overdosing can cause overload of the renal and hepatic systems in vulnerable patients [31]. These findings suggest that nutritional adequacy operates within physiological ranges; however, the consequences of excess intake are context-dependent. For example, while inadequate protein intake is associated with sarcopenia and impaired recovery, evidence of renal harm from higher protein consumption is largely confined to individuals with pre-existing CKD; randomized trials in healthy populations have not consistently demonstrated clinically significant renal impairment [38]. The strategy of moderation, nutrient quality, and individualized assessment should hence be used as a preventive measure to ensure metabolic equilibrium [45]. Figure 3 shows that excessive intake of nutrients, including vitamins, minerals, carbohydrates, fats, and proteins, can lead to various toxicities and metabolic disorders.

Nutritional therapy and clinical interventions

Nutrition as Preventive and Therapeutic Strategy

Nutrition functions as both a preventive and therapeutic modality in the management of acute and chronic disease [52]. Current nutritional therapy combines targeted dietary modification, selective supplementation, and population-level fortification, aimed at normalizing physiological homeostasis and achieving positive clinical outcomes [33]. Dietary modification remains the foundation of therapeutic intervention [53].

Large-scale trials provide evidence for balanced dietary patterns, including the Mediterranean diet, the Dietary Approaches to Stop Hypertension (DASH) diet, and plant-based dietary models, which are associated with reduced cardiovascular, metabolic, and inflammatory risk [25]. These dietary patterns exert beneficial effects partly through improved lipid profiles, reduced oxidative stress, and enhanced glycemic regulation [19]. They emphasize whole grains, legumes, fruits, vegetables, lean protein sources, and unsaturated fats while limiting processed foods and added sugars [12].

Supplementation and Fortification Strategies

Supplementation and food fortification are primarily directed at correcting micronutrient deficiencies at both individual and population levels [45]. Iron and folic acid fortification have reduced the incidence of anemia and neural tube defects, while iodine fortification has decreased rates of goitre and hypothyroidism [37]. Vitamin D and calcium fortification contribute to the maintenance of skeletal health in populations at risk.

Selective supplementation, such as omega-3 fatty acids for hypertriglyceridemia, vitamin B12 for pernicious anemia, or zinc to support wound healing, has demonstrated therapeutic benefit in specific clinical contexts [54]. However, supplementation should be guided by clinical and biochemical assessment to prevent excessive intake, nutrient imbalance, or adverse interactions [31].

Medical Nutrition Therapy and Clinical Integration

Medical nutrition therapy (MNT) is an established component of chronic disease management [43]. Personalized carbohydrate planning improves glycemic control in diabetes, controlled protein and electrolyte intake reduces metabolic burden in CKD, and enteral nutrition and parenteral nutrition ensure adequate nutrient provision when oral intake is insufficient [46].

Recent studies emphasize the role of functional foods and bioactive compounds, such as polyphenols, probiotics, and bioactive peptides, in modulating gut microbiota composition, inflammatory pathways, and oxidative balance [55]. Evidence supporting these interventions varies in strength, with mechanistic and short-term interventional data often preceding long-term outcome trials.

These developments illustrate the expanding therapeutic scope of nutrition and its potential to complement pharmacologic therapy within evidence-based clinical practice [28]. Table 3 summarizes evidence-based dietary strategies and targeted nutrient therapies that support disease prevention and clinical management.

**Table 3:** Therapeutic Nutrition Strategies, Clinical Applications, and Health Outcomes DASH: Dietary Approaches to Stop Hypertension; MNT: medical nutrition therapy

Nutritional Strategy	Clinical Condition	Mechanism of Action	Therapeutic Outcome	References
Mediterranean diet	Cardiovascular and metabolic disorders	Rich in unsaturated fats, polyphenols, and fibre, it reduces oxidative stress and inflammation	Improved lipid profile and reduced CVD risk	[19,25,45]
DASH diet	Hypertension and endothelial dysfunction	Low sodium, high potassium, calcium, and magnesium intake	Reduced blood pressure and enhanced vascular function	[25,32,37]
Plant-based diets	Metabolic and inflammatory diseases	High antioxidants and fibre; low saturated fat intake	Decreased inflammation and better metabolic control	[19,25,28]
Iron and folic acid fortification	Anemia and neural-tube defects	Enhances hemoglobin synthesis and DNA methylation	Lower anemia prevalence and reduced birth defects	[17,37,45]
Vitamin D and calcium fortification	Skeletal and endocrine disorders	Regulates calcium–phosphate metabolism and bone mineralization	Improved bone density and endocrine balance	[20,31,37]
Omega-3 fatty acids	Hypertriglyceridemia and inflammation	Produces anti-inflammatory eicosanoids and reduces triglycerides	Lowered serum triglycerides and improved cardiac function	[19,43,54]
Vitamin B12 supplementation	Pernicious anemia and neurological disorders	Supports erythropoiesis and myelin formation	Corrected anemia and neurological improvement	[29,31,54]
Zinc supplementation	Wound healing and immune repair	Promotes collagen synthesis and immune modulation	Enhanced tissue regeneration and immune response	[40,41,43]
MNT	Diabetic, renal, and critical care patients	Personalized carbohydrate, protein, and electrolyte control	Improved metabolic management and clinical outcomes	[33,43,44,46,52]
Functional foods/bioactive compounds	Gut microbiota, inflammation, oxidative stress	Polyphenols, probiotics, and peptides modulate immune and metabolic pathways	Reduced oxidative stress and led to better gut health	[19,38,55]

New trends: customized and targeted nutrition

The scientific field of nutrition has been on a path of personalization, merging molecular biology, data analytics, and behavioral science [42]. Precision nutrition aims to match the nutritional prescriptions to the genetic, metabolic, and microbiome paradigm of a particular individual, since reactions to the identical diet vary among individuals [18]. Nutrigenomics can be defined as the study of the effects of nutrients on gene expression, and nutrigenetics as the study of the effect of genetic variation on nutrient metabolism [27]. As an example, MTHFR polymorphism alters the folate metabolic rate and folate supplementation response, and FADS1 variants alter omega-3 fatty acid metabolism [31]. These discoveries can be used to provide genotype-specific dietary prescriptions that are metabolically efficient and prevent diseases [36]. Metabolomics can be used to supplement the two methods because it detects biochemical indicators of nutrient imbalance, which can be detected early and acted upon before the clinical conditions present [12]. The intestinal flora has taken centre stage as a mediator of nutrient-host interactions [25]. The intestinal flora breaks down dietary fibre, polyphenols, and amino acids to produce bioactive metabolites, including short-chain fatty acids, which control inflammation, insulin sensitivity, and lipid metabolism [30]. A lack of diversity in the gut microbiota also leads to obesity, diabetes, and autoimmune disorders, but adding probiotics, prebiotics, and high-fibre diets helps to restore microbial diversity and increase metabolic resilience [39]. Microbiome profiling as a part of the personalized nutrition approaches, therefore, further enhances precision nutrition by going beyond genetics [44]. Nowadays, personalized nutrition is actually operationalized through digital technologies [15]. Continuous glucose monitoring, wearable devices, and artificial intelligence-based dieting platforms offer personalized feedback correlating dieting with physiological reaction [48]. These systems facilitate dynamic nutrition control, facilitating proportionate intake of macronutrients and optimizing eating times to maintain homeostasis [21]. On the population level, big-data analytics assists in monitoring dietary tendencies and risk factors contributing to the development of personal and population-wide strategies [52]. A combination of genomics, metabolomics, and digital health applications is driving the transformation of population-based nutrition to personalized and data-driven nutrition [33]. Such evolution is set to enhance the quality of life, optimize therapy, and advance disease prevention [46]. Nonetheless, it will be successful depending on equitable access, provisions of privacy of data, and interdisciplinary collaboration [19]. Precision nutrition is, therefore, not only a new technology but also a new fashion of healthcare where the focus is on personalization and sustainability [50].

Limitations and future directions

Despite substantial investigation into the clinical roles of macronutrients and micronutrients, several limitations remain within the current evidence base and within the scope of this review.

Limitations of the Evidence Base

A considerable proportion of nutrition research is geographically or demographically restricted, limiting external validity across diverse populations. Many studies rely on observational designs and self-reported dietary assessments, which are vulnerable to recall bias, measurement error, and residual confounding. Although such studies provide valuable associative insights, causal inference remains limited without long-term randomized controlled trials.

In addition, much of the literature evaluates isolated nutrients rather than whole dietary patterns or interactive nutrient effects. Randomized trials examining combined macronutrient-micronutrient interactions remain comparatively limited. Substantial heterogeneity exists in nutrient definitions, dosage ranges, supplementation formulations, baseline dietary exposures, and clinical outcome measures. This variability constrains direct comparability across studies and may contribute to inconsistent findings.

Differences in nutrient bioavailability, genetic polymorphisms, microbiome composition, and lifestyle behaviors further complicate interpretation. These factors introduce biological variability that may influence both effect size and reproducibility. Accordingly, while pooled evidence provides meaningful direction, conclusions must be interpreted within the context of methodological and biological heterogeneity rather than assumed to be universally generalizable.

Methodological Limitations of this Review

This was a narrative review and did not include a formal risk-of-bias assessment or graded quality appraisal of included studies. As a result, the strength of evidence varies across the cited literature, and conclusions reflect qualitative synthesis rather than structured evidence grading.

Given the broad scope, some conclusions are drawn upon prior systematic reviews and meta-analyses in addition to primary studies. Although this approach enables comprehensive integration, it may also inherit limitations present in earlier syntheses.

Limitations and Current Constraints of Precision Nutrition

Precision nutrition is presented as a promising future direction; however, its current implementation remains constrained. Large-scale clinical validation trials are limited, long-term outcome data are incomplete, and questions remain regarding scalability, cost-effectiveness, and equitable access. Genetics- and omics-based personalization strategies, while mechanistically compelling, have not yet been consistently translated into standardized clinical protocols.

Future Research Directions

Future research would benefit from multidimensional, data-driven frameworks integrating genomics, metabolomics, and microbiomics to clarify nutrient-gene and nutrient-microbiome interactions. Improvements in dietary assessment technologies, including digital tracking and real-time metabolic analytics, may reduce measurement errors. Longitudinal and well-powered interventional trials are needed to strengthen causal inference and refine context-specific dietary recommendations.

Global nutrition policies should integrate sustainability, cultural adaptability, and equitable access to ensure that advances in nutritional science translate into population-level benefit without exacerbating health disparities.

## Conclusions

This review integrates current evidence on the clinical relevance of macronutrients and micronutrients within an interconnected metabolic framework. The synthesis indicates that nutritional imbalance, whether through deficiency, excess, or disproportionate distribution, is consistently associated with adverse metabolic and clinical outcomes. At the same time, the strength of evidence varies across domains. While correction of established deficiencies and substitution-based dietary strategies are supported by relatively robust data, broader claims regarding optimal macronutrient ratios, universal supplementation, or disease modification require cautious interpretation given heterogeneity in study design, population characteristics, and outcome measures.

Emerging areas such as precision nutrition, nutrigenomics, and microbiome-informed interventions provide mechanistic insights into the inter-individual variability in dietary response; however, large-scale clinical validation, long-term outcome evidence, and equitable implementation strategies remain limited. Current evidence therefore supports a context-dependent, pattern-based approach to nutrition that emphasizes balanced dietary structures and targeted interventions within defined clinical indications. Further rigorously designed interventional research is required before systems-oriented and personalised strategies can be fully integrated into routine clinical practice.
